# Supplementary material for: Designing receptor agonists with enhanced pharmacokinetics by grafting macrocyclic peptides into fragment crystallizable regions
Source: Nat Biomed Eng. 2022 Nov 7;7(2):164–76. doi: 10.1038/s41551-022-00955-6 (PMC9991925; doi:10.1038/s41551-022-00955-6)
Supplement: Supplementary file 1 — Supplementary figures. [file 41551_2022_955_MOESM1_ESM.pdf]

nature biomedical engineering

Article

https://doi.org/10.1038/s41551-022-00955-6

Designing receptor agonists with enhanced pharmacokinetics by grafting macrocyclic peptides into fragment crystallizable regions

In the format provided by the authors and unedited

a

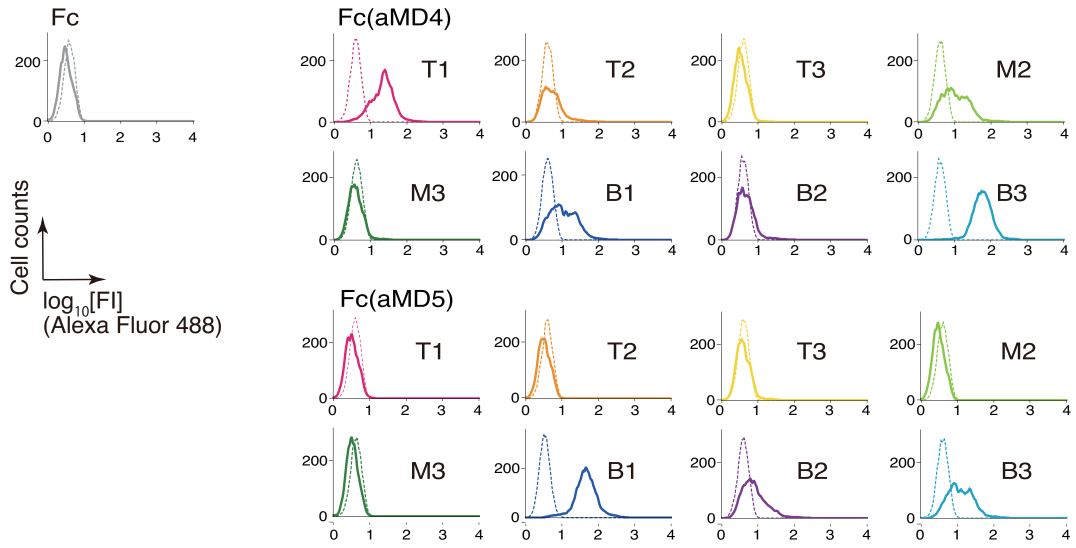

b

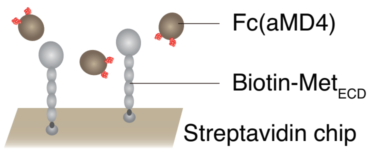

	k_a (10^5/Ms)	k_d (10^{-2}/Ms)	K_D (nM)
aMD4-dimer	14.00	0.11	0.9
Fc(aMD4)T1	2.27	1.72	75.8
Fc(aMD4ds)T1	0.62	0.46	73.4
Fc(aMD4)T2	0.12	0.47	401.0
Fc(aMD4)T3	0.28	1.84	652.4
Fc(aMD4)M2	1.37	2.58	18.9
Fc(aMD4)B1	7.08	0.77	10.8
Fc(aMD4)B2	3.54	2.80	79.1
Fc(aMD4)B3	3.26	0.13	4.0
Fc(aMD4ds)B3	3.56	0.13	3.7

Supplementary Fig. 1 | Target loop position of lasso-grafting determines affinity to Met. a, Binding of Fc and Fc(aMD4/5) to Met-knockout CHO cells (dashed lines) or Met-reconstituted CHO cells (bold lines) determined by flow cytometry indicating the correlation of Met binding and Met agonist activities (Fig. 1b, c). FI, fluorescence intensity. **b**, Binding kinetics of aMD4-dimer peptide or Fc(aMD4) to Met ectodomain (Met_{ECD}) as determined by surface plasmon resonance. Representative results calculated with five concentrations of analytes are shown. The values for aMD4-dimer peptide were taken from a previous report[40]. The association rates of Fc(aMD4) were 2.0–116.7-fold slower than aMD4-dimer peptide ($k_a = 7.08$–0.12×10^5/Ms vs. $k_a = 14.0 \times 10^5$/Ms, respectively), likely due to their larger molecular size. However, the dissociation rates of Fc(aMD4) were comparable to that of the aMD4-dimer peptide ($k_d = 0.13$–2.58×10^{-2}/Ms vs. 0.11×10^{-2}/Ms, respectively), indicating that the stability of the complex between Met_{ECD} and the peptide moiety was maintained even when presented in the context of Fc loop.

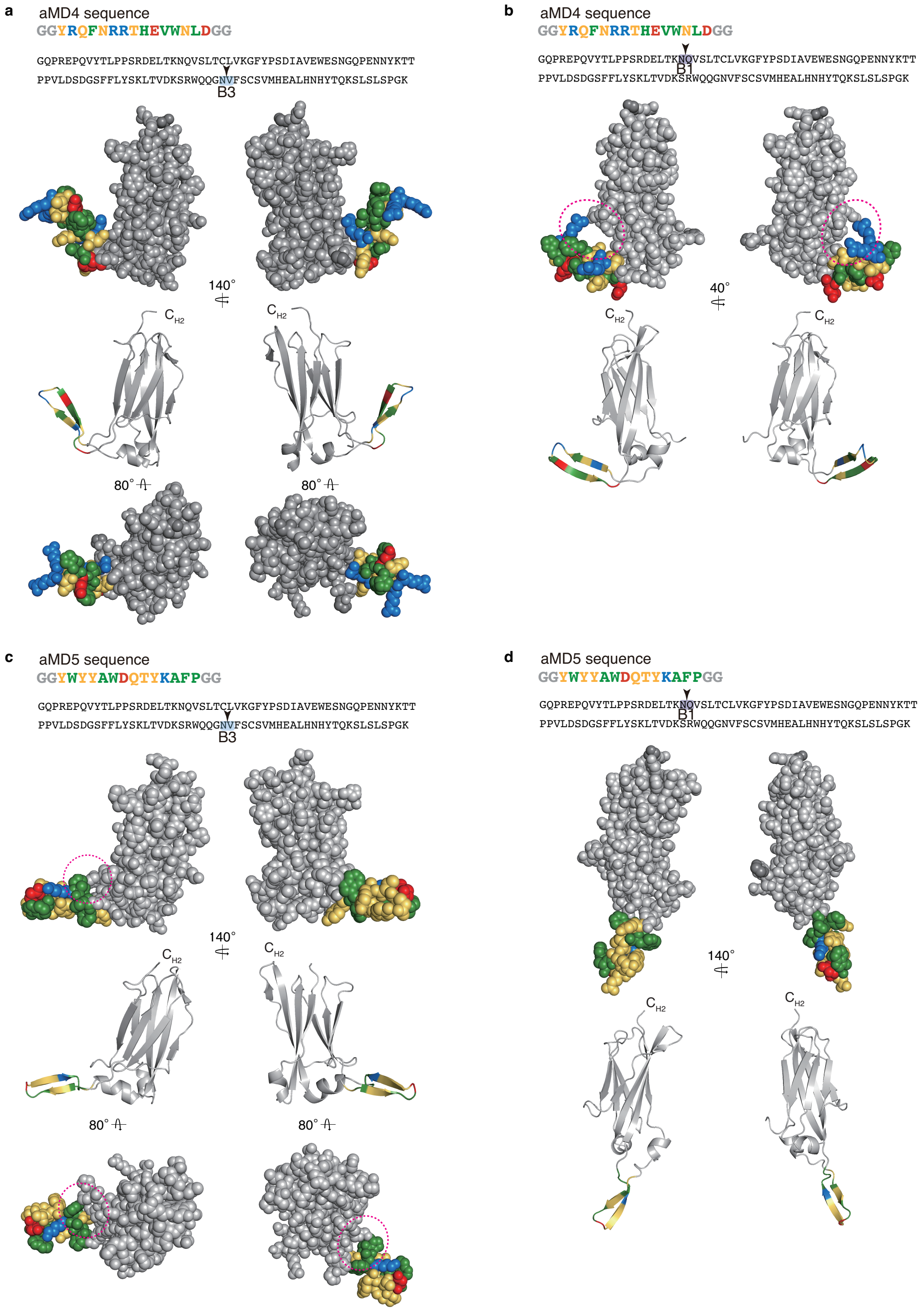

Supplementary Fig. 2 | Structures of the CH3 domain of Fc with aMD4 or aMD5 inserted in the B1 or B3 loop predicted by ColabFold. Non-polar amino acids of aMD4 and aMD5 are shown in green, polar amino acids in yellow, acidic amino acids in red, and basic amino acids in blue. Note the different spatial arrangements of the inserted peptides in each case. The magenta dashed lines indicate that the area of peptide moiety that may structurally interfered by the Fc backbone.

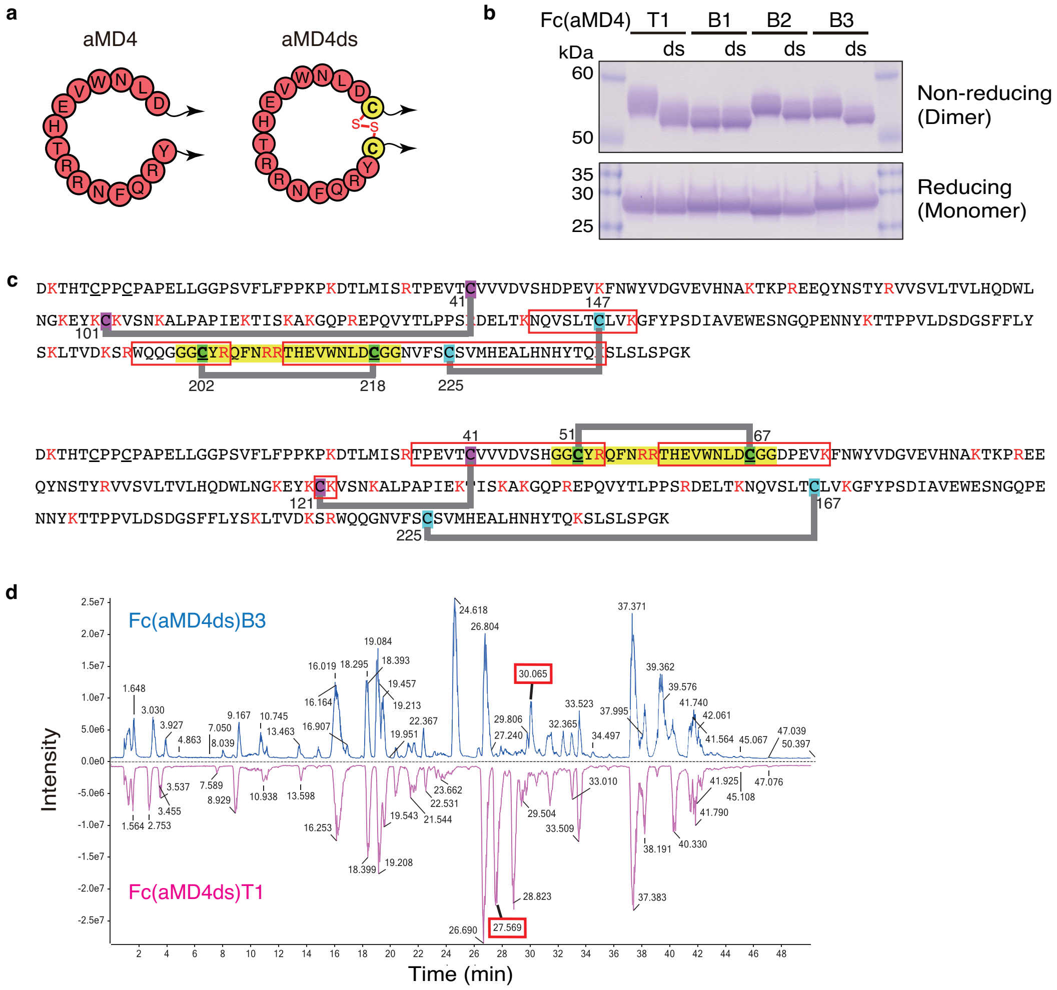

e

Retention Time	Theoretical Mono m/z	Observed Mono m/z	Error (ppm)	Score	Charge	XIC Area	Sequence	Disulfide Bonds
30.05	1111.3106	1111.3099	-0.6	7.568	5	1.24E+05	NQVSLTCLVK; WQQGGCYR; THEVWNLDCGGNVFSCSVM HEALHNHYTQK	202=218, 147=225
30.07	926.26	926.2595	-0.5	7.098	6	8.75E+05	NQVSLTCLVK; WQQGGCYR; THEVWNLDCGGNVFSCSVM HEALHNHYTQK	202=218, 147=225

Retention Time	Theoretical Mono m/z	Observed Mono m/z	Error (ppm)	Score	Charge	XIC Area	Sequence	Disulfide Bonds
27.57	793.5612	793.562	1	11.878	5	1.06E+07	TPEVTCVVVDVS HGGCYR; THEVWNLDCGG DPEVK; CK	121=41, 67=51
27.57	496.3535	496.3536	0.4	7.098	8	4.33E+06	TPEVTCVVVDVS HGGCYR; THEVWNLDCGG DPEVK; CK	121=41, 67=51
27.57	991.6997	991.7002	0.5	12.719	4	1.68E+06	TPEVTCVVVDVS HGGCYR; THEVWNLDCGG DPEVK; CK	121=41, 67=51

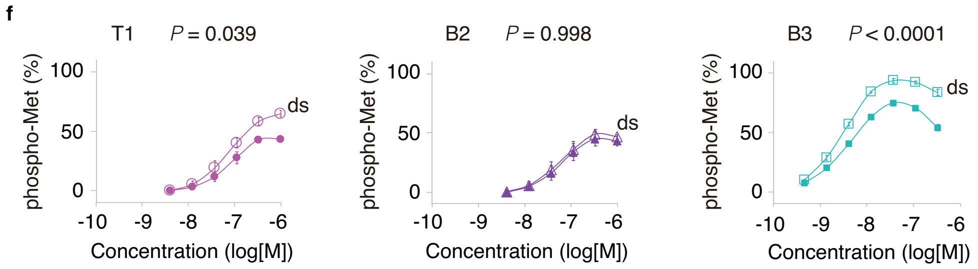

Supplementary Fig. 3 | Met agonist activity of disulfide-linked aMD4 derivatives. a, The pharmacophore sequence of aMD4 with cysteine residues at both ends (aMD4ds) was inserted into the loops of human IgG1 Fc protein. **b**, SDS-PAGE analysis of Fc(aMD4) and Fc(aMD4ds) under non-reducing (above) and reducing conditions (below). Under reducing conditions, Fc(aMD4ds) variants showed marginally lower electrophoretic mobilities than Fc(aMD4) variants, due to the slightly increased molecular weight [Fc(aMD4): 55,584 Da, Fc(aMD4ds): 55,996 Da] caused by two cysteine insertions per chain. However, under non-reducing conditions, Fc(aMD4ds) other than B1 position showed greater electrophoretic mobilities than Fc(aMD4), strongly suggesting the presence of an additional disulfide-linkage. **c–e**, LC-MS/MS analysis of tryptic digests of Fc(aMD4)T1 or Fc(aMD4)B3 confirmed disulfide bonds at both ends of aMD4 sequence. **c**, Amino acid sequences of Fc(aMD4ds)B3 (above) and Fc(aMD4ds)T1 (below). Peptides derived from the disulfide-linked aMD4 sequence after trypsin digestion are indicated by red boxes. Disulfide bonds are indicated by gray lines. Arginine and lysine recognized by trypsin are shown in red. **d–e**, Comparison mirror plots of LC chromatograms from tryptic digests of Fc(aMD4ds)B3 (above) and Fc(aMD4ds)T1 (below). The total ion current chromatograms (TICs) are shown. The characteristic peaks with retention times of 30.1 min [Fc(aMD4ds)B3] and 27.6 min [Fc(aMD4ds)T1] are derived from the disulfide-linked aMD4 sequence (red boxes in **c**) as indicated by the amino acid sequences and the sites of disulfide linkage analyzed by MS and MS/MS spectra shown in **e**. The peptides identified by the analysis covered 85% of Fc(aMD4ds)B3 or 87% of Fc(aMD4ds)T1. There was a complete absent of aMD4ds peptides without disulfide bonds. **f**, The dsT1 and dsB3 variants showed enhanced cellular Met activation, compared with controls. The results are presented as the mean ± S.E.M. (n = 3, independent experiments; two-way ANOVA) of percentage (%) of phospho-Met relative to maximum Met phosphorylation induced by 1.1 nM HGF.

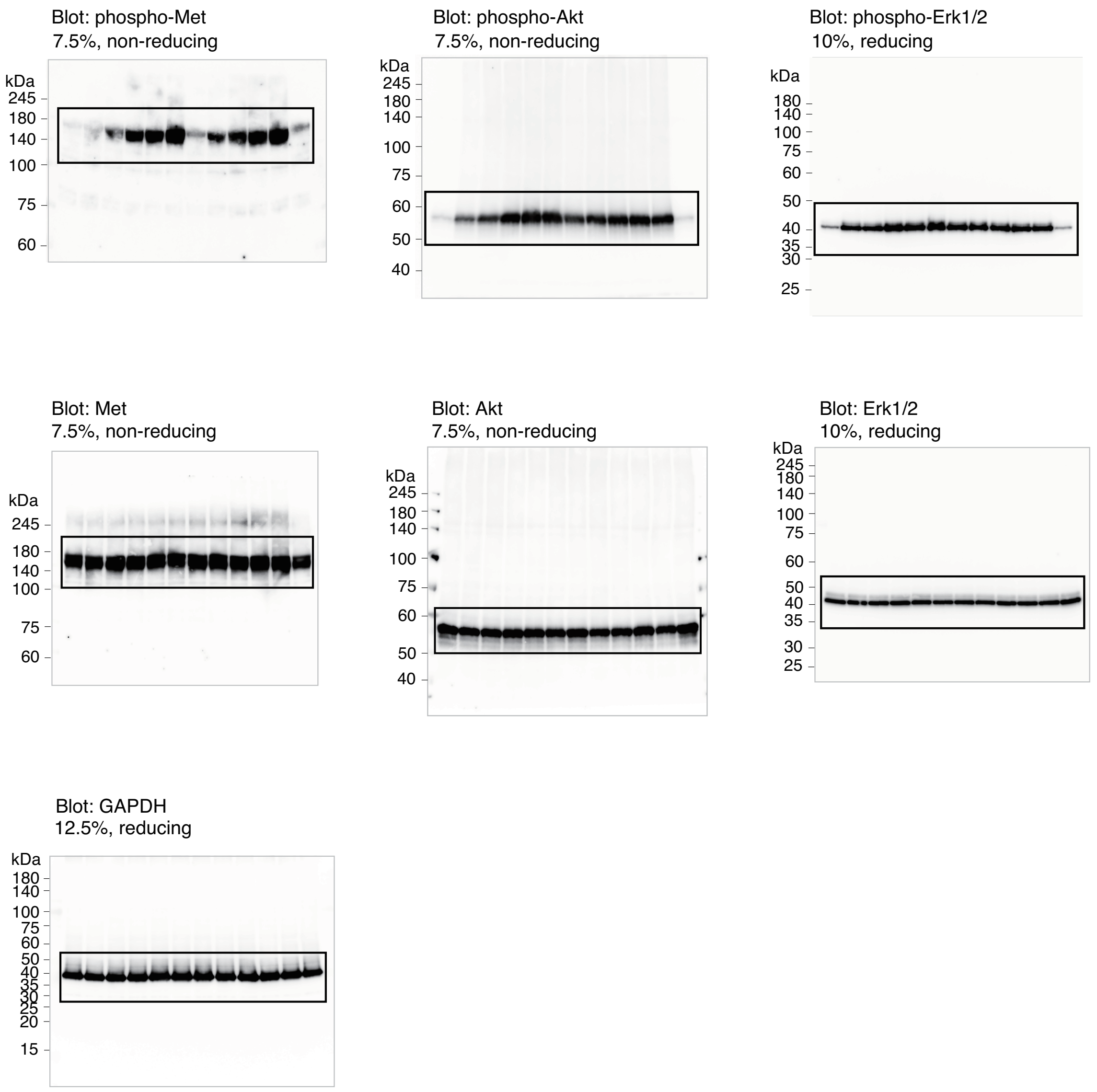

Supplementary Fig. 4 | Uncropped images of Western blots in Fig. 2b.

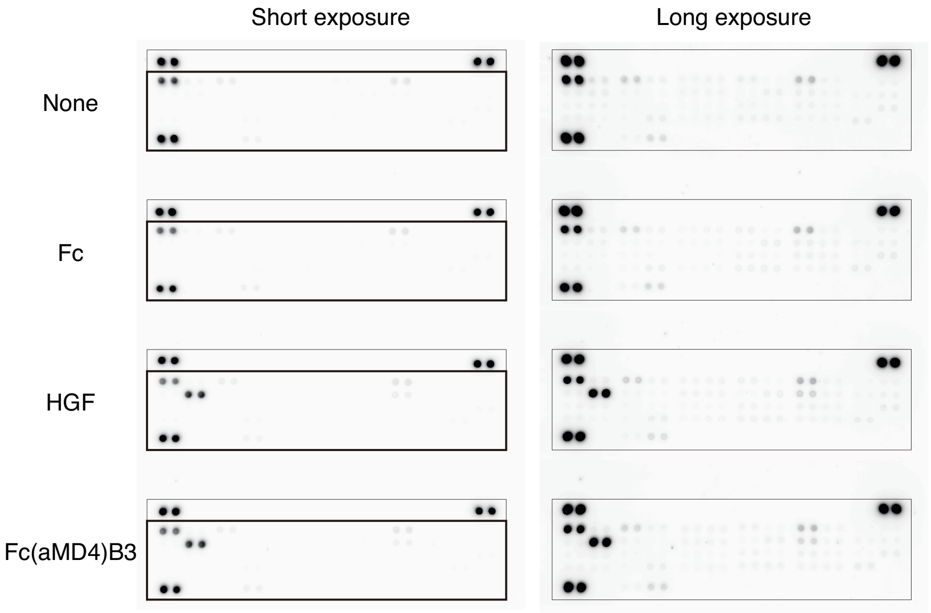

Posi Ctrl											Posi Ctrl
EGFR	ErbB2	ErbB3	ErbB4	FGFR1	FGFR2α	FGFR3	FGFR4	Insulin R	IGF-R	Axl	Dtk
Mer	Met	MSPR	PDGFRα	PDGFRβ	SCFR	Flt-3	M-CSFR	c-Ret	ROR1	ROR2	Tie-1
Tie-2	TrkA	TrkB	TrkC	VEGFR1	VEGFR2	VEGFR3	MuSK	EphA1	EphA2	EphA3	EphA4
EphA6	EphA7	EphB1	EphB2	EphB4	EphB6	ALK	DDR1	DDR2	EphA5	EphA10	
Posi Ctrl		EphB3	RYK								Neg Ctrl

Supplementary Fig. 5 | Uncropped images of Western blots in Fig. 2c.

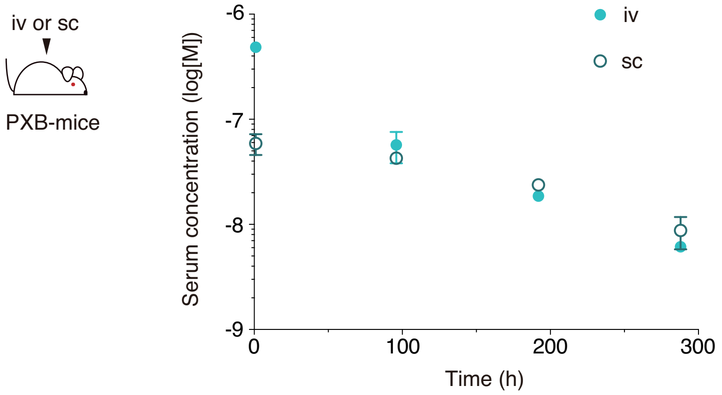

Supplementary Fig. 6 | Serum concentration of Fc(aMD4)B3 in PXB-mice. Chimeric mice with humanized liver (PXB-mice) were given a single iv injection *via* the tail vein or sc injection of Fc(aMD4)B3 at 5 mg/kg, and serum concentrations at various time points were determined by ELISA. The results are presented as the mean ± S.E.M. (*n* = 3 mice per group).

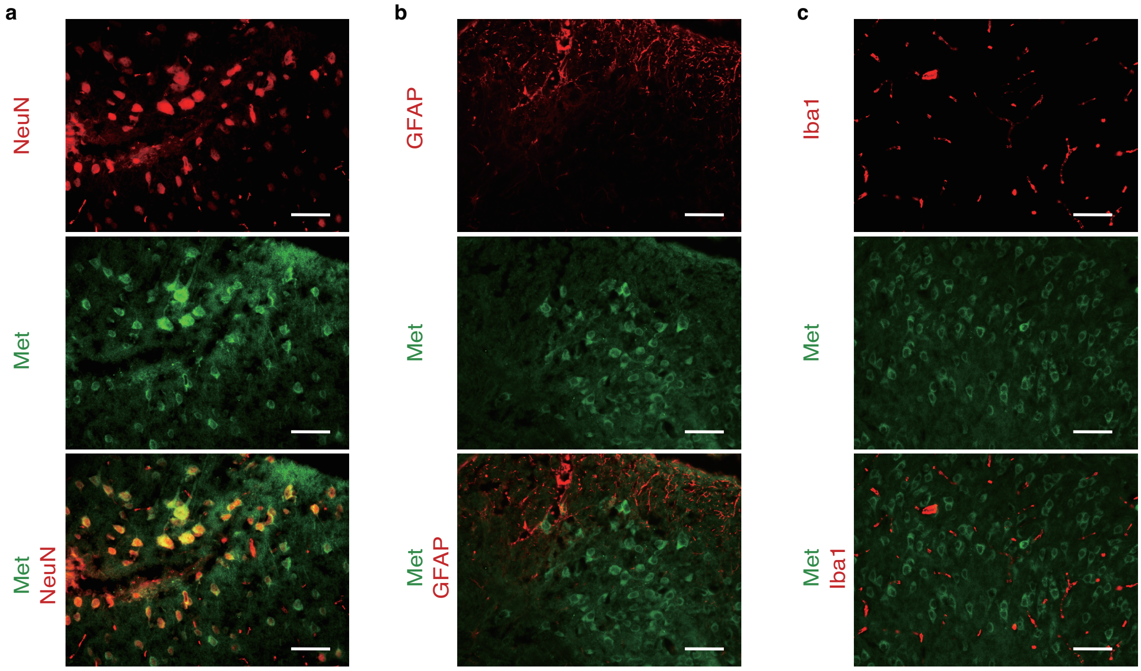

Supplementary Fig. 7 | Met expression in mouse brain sections. Note the strong staining for Met in NeuN-positive neurons (**a**), but not in GFAP-positive astrocytes (**b**) and Iba1-positive microglia cells (**c**). Scale bar: 50 µm.

a

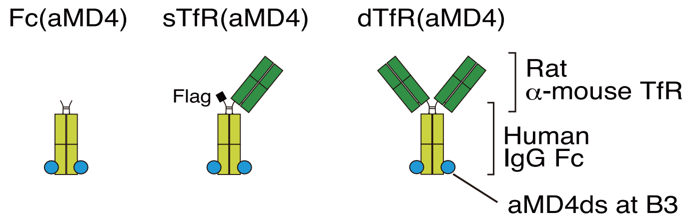

b

Rat α-mouse TfR Heavy Chain

Signal peptide → VH

MDWTWRVFCLLAVAPGAHS EVQLVESGGGLVQPGNSLTLSCVASGFTFSNYGMHWIRQAPKKGLEWIAMIYYDSS

CH1

KMNYADTVKGRFTISRDNSKNTLYLEMNSLRSEDTAMYYCAVPTSHYVVDVWGQGVSVTVSS AKTTPPSVYPLA

PGSAAQTNSMVTLGCLVKGYFPEPVTVTWNSGSLSSGVHTFPAVLQSDLYTLSSSVTVPSSPRPSETVTCNVAHP

Human IgG Fc →

ASSTKVDKKI DKTHTCPPCPAPELLGGPSVFLFPPKPKDTLMISRTPEVTCVVVDVSHEDPEVKFNWYVDGVEVHN

AKTKPREEQYNSTYRVVSVLTVLHQDWLNGKEYKCKVSNKALPAPIEKTISKAKGQPREPQVYTLPPSRDELTKN

aMD4ds

QVSLTCLVKGFYPSDIAVEWESNGQPENNYKTTPPVLDSDGSFFLYSKLTVDKSRWQQGG**CYRQFNRRTHEVWNLD**

CGGNVFSCSVMHEALHNHYTQKSLSLSPGK

c

Rat α-mouse TfR Light Chain

Signal peptide → VL

METPAQLLFLLLLWLPDTTG DIQMTQSPASLSASLEEIVTITCQASQDIGNWLAWYQQKPGKSPQLLIYGATSLAD

CL

GVPSRFSGSRSGTQFSLKISRVQVEDIGIYYCLQAYNTPWTFGGGTKLELKR ADAAPTVSIFPPSSEQLTSGGAS

VVCFLNNFYPKDINVKWKIDGSERQNGVLNSWTDQDSKDSTYSMSSTLTLTKDEYERHNSYTCEATHKTSTSPIVKS

FNRGEC

Supplementary Fig. 8 | Construction and purification of anti-TfR antibodies lasso-grafted with aMD4ds at the B3 site. a, Schematic representations of Fc(aMD4), dTfR(aMD4), and sTfR(aMD4). All variants were grafted with aMD4ds at the B3 loop of human IgG Fc. Rat anti-mouse transferrin receptor Fab was fused with Fc(aMD4). **b–c**, Amino acid sequence of (**b**) the chimeric heavy chain of rat anti-mouse TfR Fab and human IgG Fc grafted with aMD4ds at B3 or (**c**) the light chain of rat anti-mouse TfR.

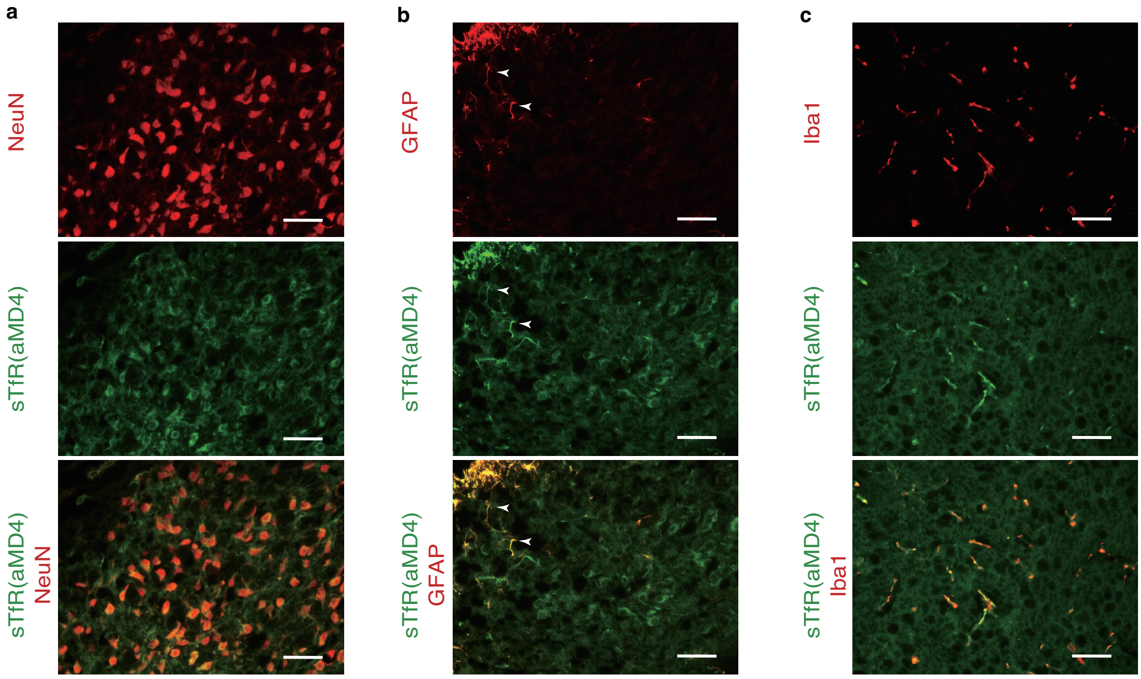

Supplementary Fig. 9 | sTfR(aMD4) co-localizes with (a) NeuN-, (b) GFAP-, or (c) Iba1-positive cells in brain sections from mice at 24 h after a single injection of sTfR(aMD4) via the tail vein. Note the broad staining for sTfR(aMD4) in the brain parenchyma and localization around NeuN-positive neuronal cells, GFAP-positive astrocytes (arrows), and Iba1-positive microglia cells. Scale bar: 50 μm.

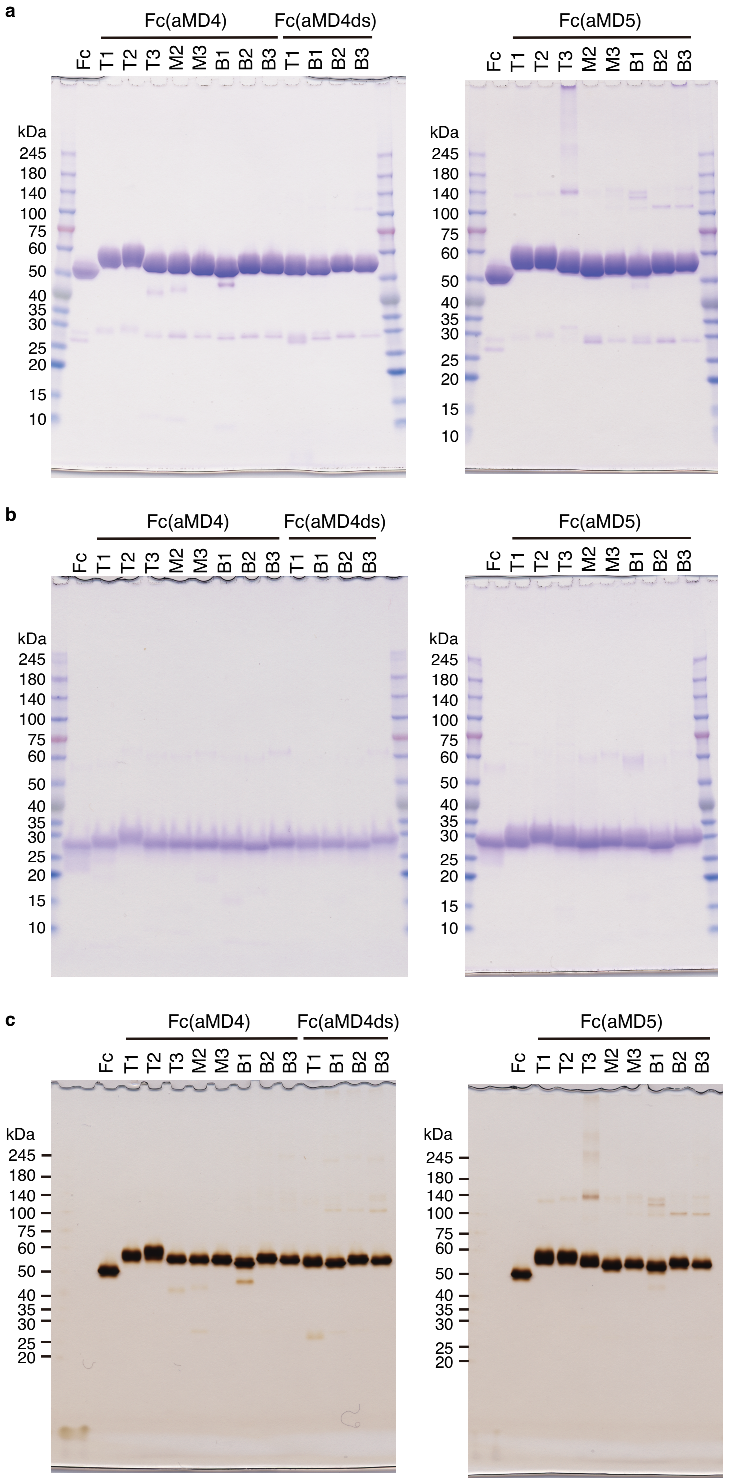

d

	Purity (%)
Fc	92.5
Fc(aMD4)T1	96.4
Fc(aMD4)T2	95.0
Fc(aMD4)T3	90.2
Fc(aMD4)M2	89.1
Fc(aMD4)M3	96.6
Fc(aMD4)B1	89.7
Fc(aMD4)B2	95.3
Fc(aMD4)B3	95.2
Fc(aMD4ds)T1	91.0
Fc(aMD4ds)B1	91.8
Fc(aMD4ds)B2	92.6
Fc(aMD4ds)B3	92.4
Fc	93.0
Fc(aMD5)T1	97.6
Fc(aMD5)T2	96.3
Fc(aMD5)T3	86.1
Fc(aMD5)M2	94.0
Fc(aMD5)M3	95.3
Fc(aMD5)B1	90.8
Fc(aMD5)B2	91.4
Fc(aMD5)B3	93.3

Supplementary Fig. 10 | Purified Fc(aMD4), Fc(aMD4ds), and Fc(aMD5) used in the assay. a–c, Fc(aMD4), Fc(aMD4ds), and Fc(aMD5) were analyzed by SDS-PAGE under non-reducing (**a**, **c**) or reducing (**b**) conditions and stained with Coomassie brilliant blue (**a**, **b**) or silver stained (**c**). **d**, Purity was analyzed using ImageJ on images presented in **a**.

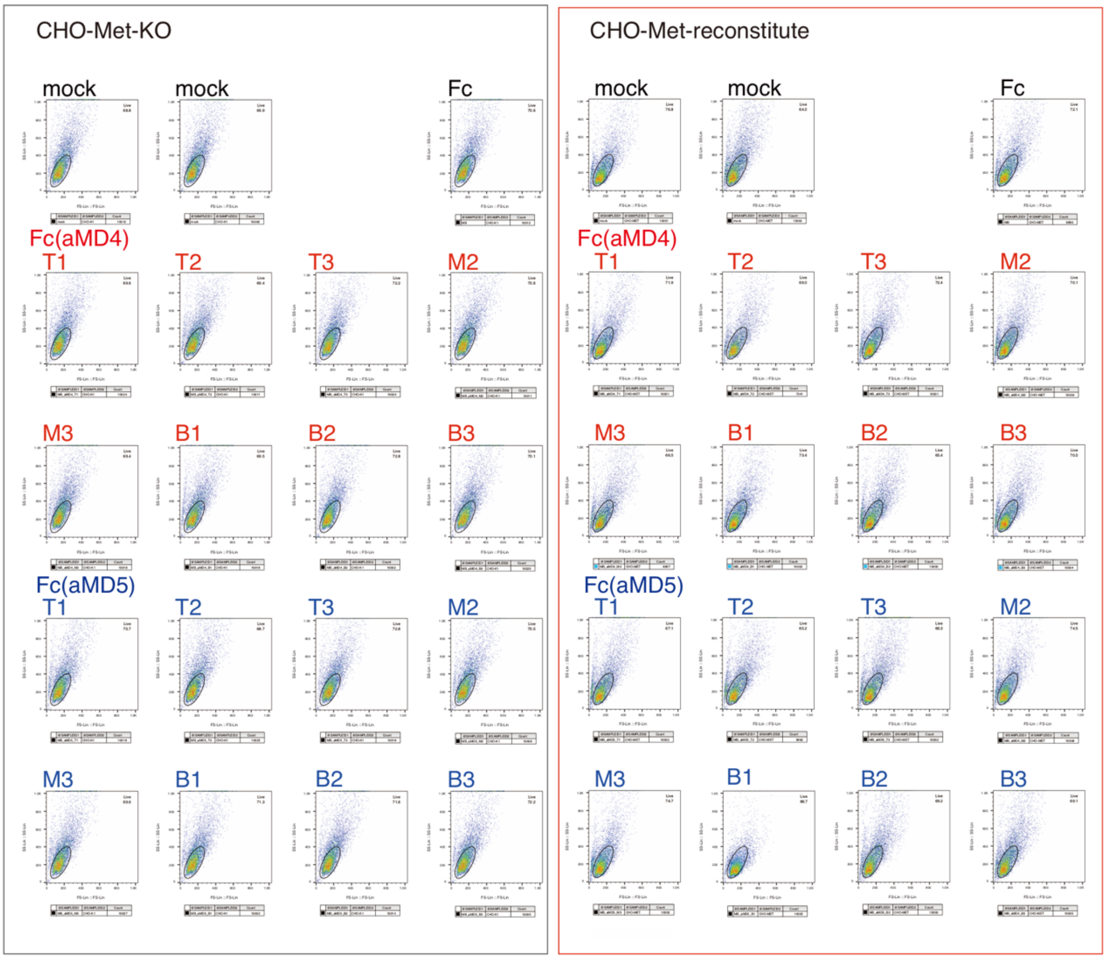

Supplementary Fig. 11 | Flow cytometry gating strategies. Scatterplots of forward scatter *vs*. side scatter showing live cell gating (black ovals) for each histogram panel in Supplementary Fig. 1a. The gate was set to include all cell populations, but excluding debris and dead cells.
