# Supplementary material for: Designing receptor agonists with enhanced pharmacokinetics by grafting macrocyclic peptides into fragment crystallizable regions
Source: Nat Biomed Eng. 2022 Nov 7;7(2):164–76. doi: 10.1038/s41551-022-00955-6 (PMC9991925; doi:10.1038/s41551-022-00955-6)
Supplement: Supplementary file 2 — Reporting Summary [file 41551_2022_955_MOESM2_ESM.pdf]

nature portfolio

Corresponding author(s): Katsuya Sakai, Junichi Takagi, and Kunio Matsumoto

Last updated by author(s): Sep 19, 2022

Reporting Summary

Nature Portfolio wishes to improve the reproducibility of the work that we publish. This form provides structure for consistency and transparency in reporting. For further information on Nature Portfolio policies, see our Editorial Policies and the Editorial Policy Checklist.

Statistics

For all statistical analyses, confirm that the following items are present in the figure legend, table legend, main text, or Methods section.

n/a	Confirmed	
☐	☒	The exact sample size (*n*) for each experimental group/condition, given as a discrete number and unit of measurement
☐	☒	A statement on whether measurements were taken from distinct samples or whether the same sample was measured repeatedly
☐	☒	The statistical test(s) used AND whether they are one- or two-sided<br>*Only common tests should be described solely by name; describe more complex techniques in the Methods section.*
☒	☐	A description of all covariates tested
☒	☐	A description of any assumptions or corrections, such as tests of normality and adjustment for multiple comparisons
☐	☒	A full description of the statistical parameters including central tendency (e.g. means) or other basic estimates (e.g. regression coefficient) AND variation (e.g. standard deviation) or associated estimates of uncertainty (e.g. confidence intervals)
☐	☒	For null hypothesis testing, the test statistic (e.g. *F*, *t*, *r*) with confidence intervals, effect sizes, degrees of freedom and *P* value noted<br>*Give P values as exact values whenever suitable.*
☒	☐	For Bayesian analysis, information on the choice of priors and Markov chain Monte Carlo settings
☒	☐	For hierarchical and complex designs, identification of the appropriate level for tests and full reporting of outcomes
☒	☐	Estimates of effect sizes (e.g. Cohen's *d*, Pearson's *r*), indicating how they were calculated

Our web collection on statistics for biologists contains articles on many of the points above.

Software and code

Policy information about availability of computer code

Data collection

Chemiluminescence and absorbance were measured using an ARVO MX plate reader (Perkin Elmer).
Cell-surface fluorescence was measured using an EC800 system (Sony).
SPR was performed using Biacore T200 instrument (Cytiva) or Biacore 3000 instrument (Cytiva).
Western blotting images were captured using a FUSION-SOLO.6S.EDGE (VIRVER).
RNA-seq was performed using a DNBSEQ-G400 sequencer (MGI TECH).
Immunohistochemical staining images were captured using a BZ-X810 (Keyence).
LC-MS/MS was performed using a Nexera X2 UHPLC system (Shimadzu corporation) connected to a SCIEX TripleTOF 6600 system (AB SCIEX),
HS-AFM images were collected using Igor Pro Ver. 6.3.6.0. (WaveMetrics).

Data analysis

Prism 6.0d (GraphPad) was used graphing and statistical analysis.
Binding kinetics were analysed using Biacore T200 evaluation software version 3.2 (GE Healthcare) or BIAevaluation software version 4.1 (GE Healthcare).
HS-AFM images were analysed using Igor Pro Ver. 6.3.6.0. (WaveMetrics).
Flow-cytometry data were analysed by FlowJo software version 10.6.1.
The LC-MS raw data were processed using SCIEX BioPharmaView software (SCIEX).
RNA-seq data were analysed using cutadapt (ver. 1.9.1), sickle (ver 1.33), hisat2 software (ver. 2.2.0),and featureCounts (ver. 2.0.0). DEG analysis was performed using DEGES in TCC (ver. 1.18.0) and DESeq (ver. 1.30.0).
HS-AFM images were analysed using Igor Pro Ver. 6.3.6.0. (WaveMetrics).
Structures of Fc with aMD4 or aMD5 were predicted by ColabFold, AlphaFold2 using MMseqs2.
CBB staining was quantified using IMAGEJ.JS.

For manuscripts utilizing custom algorithms or software that are central to the research but not yet described in published literature, software must be made available to editors and reviewers. We strongly encourage code deposition in a community repository (e.g. GitHub). See the Nature Portfolio guidelines for submitting code & software for further information.

Data

Policy information about availability of data

All manuscripts must include a data availability statement. This statement should provide the following information, where applicable:

- Accession codes, unique identifiers, or web links for publicly available datasets
- A description of any restrictions on data availability
- For clinical datasets or third party data, please ensure that the statement adheres to our policy

The main data supporting the findings of this study are available within the Article and its Supplementary Information. The RNA-seq data are available at the DDBJ Sequence Read Archive under accession numbers DRA014557 (hepatocyte spheroids) and DRA014558 (livers of PXB-mice). Source data for the figures are provided with this paper. The raw data generated during the study are available from the corresponding authors on reasonable request.

Field-specific reporting

Please select the one below that is the best fit for your research. If you are not sure, read the appropriate sections before making your selection.

☒ Life sciences ☐ Behavioural & social sciences ☐ Ecological, evolutionary & environmental sciences

For a reference copy of the document with all sections, see nature.com/documents/nr-reporting-summary-flat.pdf

Life sciences study design

All studies must disclose on these points even when the disclosure is negative.

Sample size	No statistical method was used to predetermine sample sizes. Sample sizes were chosen to establish statistical significance on the basis of similar experiments reported in the literature or of data from pilot experiments. Sample sizes were chosen as large as practically possible, and adequate statistics have been applied.
Data exclusions	No data were excluded from the analyses.
Replication	Reproducibility was tested through multiple inter-experimental and intra-experimental replicates, as described in the paper per each experiment.
Randomization	Mice were randomized into treatment groups.<br>Each biochemical experiment in this study was rationally designed. Samples were not randomized for these experiments.
Blinding	Blinding was not used, because the design, execution and analysis of certain experiments was in many cases performed by a single investigator. This was necessary for data analysis and to minimize potential transposition error. For RNA-seq and LC-MS/MS analyses, sample preparation, and data acquisition and analysis were outsourced, without prior knowledge of the results.

Reporting for specific materials, systems and methods

We require information from authors about some types of materials, experimental systems and methods used in many studies. Here, indicate whether each material, system or method listed is relevant to your study. If you are not sure if a list item applies to your research, read the appropriate section before selecting a response.

Materials & experimental systems

n/a	Involved in the study	
☐	☒	Antibodies
☐	☒	Eukaryotic cell lines
☒	☐	Palaeontology and archaeology
☐	☒	Animals and other organisms
☒	☐	Human research participants
☒	☐	Clinical data
☒	☐	Dual use research of concern

Methods

n/a	Involved in the study	
☒	☐	ChIP-seq
☐	☒	Flow cytometry
☒	☐	MRI-based neuroimaging

Antibodies

Antibodies used	Anti-phosphorylated Erk1/2 (T202/Y204) (1:1,000 dilution, D13.14.4E, Cell Signaling Technology).<br>Anti-Erk1/2 (1:1,000 dilution, 137F5, Cell Signaling Technology).<br>Anti-phosphorylated Akt (S473) antibody (1:1,000 dilution, D9E, Cell Signaling Technology).<br>Anti-Akt antibody (1:1,000 dilution, 11E7, Cell Signaling Technology).<br>Anti-GAPDH antibody (1:1,000 dilution, 14C10, Cell Signaling Technology).

Anti-Met antibody (Immunoprecipitation, 1 μg for 200 μl lysates, D-4, Santacruz).
Anti-Met antibody (Western bloting, 1:1,000 dilution, D1C2, Cell Signaling Technology).
Abti-phosphorylated Met antibody (Y1234/Y1235) (1:1,000 dilution, D26, Cell Signaling Technology).
Abti-phosphorylated Met antibody (Y1003) (Western bloting, 1:1,000 dilution, 13D11, Cell Signaling Technology).
Abti-phosphorylated Met antibody (Y1349) (Western bloting, 1:1,000 dilution, 130H2, Cell Signaling Technology).
HRP-conjugated anti-rabbit Immunoglobulin antibody (1:2000 dilution for western blotting, 1:1000 dilution for cellular Met phosphorylation, #P0448, Dako).
HRP-conjugated anti-mouse Immunoglobulin antibody (1:2000 dilution for western blotting, #P0447, Dako).
Alexa Fluor 488-labeled anti-human-IgG used for flow cytometry (1:400 dilution, #A11013, ThermoFisher Scientific).
HRP-conjugated anti-human IgG Fc antibody used for ELISA (0.5 μg/ml, 100 μl/well, Bethyl Laboratories).
Anti-human IgG Fc antibody used for IHC (1: 500 dilution, Bethyl Laboratories).
Anti-mouse NeuN antibody used for IHC (1: 500 dilution, Millipore).
Alexa Fluor 488-conjugated anti-goat secondary antibody used for IHC (1:200 dilution, Thermo Fisher Scientific).
Alexa Fluor 594-conjugated anti-mouse secondary antibody used for IHC (1:200 dilution, Thermo Fisher Scientific).
Anti-human HGF rabbit polyclonal antibody for ELISA (10 μg/mL for coating, 2 μg/mL for detection, In house prepared).
Anti-BrdU antibody used for IHC (3 μg/ml, ab6326, Abcam).
Alexa Fluor 488-conjugated anti-rat secondary antibody used for IHC (1 μg/ml, ab150157, Abcam).
Anti-PA tag antibody (NZ-1) (150 μl NZ1 Sepharose for pulldown, FUJIFILM Wako).
Anti-GFAP antibody used for IHC (1:500 dilution, GA5, Cell Signaling Technology).
Anti-Iba1 antibody used for IHC (1:500 dilution, GT10312, GeneTex).

Validation

All the antibodies were optimized and validated (per assay and species) by the supplier. Information on any validation statements can be found on the manufacturer's website.

The anti-human HGF rabbit polyclonal antibody for ELISA was prepared as described in; Suzuki, Y. et al. Inhibition of Met/HGF receptor and angiogenesis by NK4 leads to suppression of tumor growth and migration in malignant pleural mesothelioma. Int J Cancer. 127, 1948-1957 (2010), and was characterized via ELISA, as described in Jangphattananont, N. et al. Distinct Localization of Mature HGF from its Precursor Form in Developing and Repairing the Stomach. Int. J. Mol. Sci. 20 (2019).

The anti-PA tag antibody NZ-1 was characterized in Fujii, Y. et al. PA tag: A versatile protein tagging system using a super high affinity antibody against a dodecapeptide derived from human podoplanin. Protein. Expres. Purif. 95, 240-247 (2014).

Eukaryotic cell lines

Policy information about cell lines

Cell line source(s)

EHMES-1 was provided by Dr. Hamada (Ehime University, Ehime, Japan). EHMES-1 was established as described in Yokoyama, A. et al. Origin of heterogeneity of interleukin-6 (IL-6) levels in malignant pleural effusions. Oncol Rep. 1,507-511 (1994).
CHO-K1 was obtained from ATCC (RRID:CVCL_0214).
Expi293F was obtained from Thermo Fisher (RRID: CVCL_D615).
Met-knockout CHO-K1 cells and Met-reconstituted CHO-K1 cells were described in ref. 27.

Authentication

All cell lines were expanded upon initial receipt to create large stocks of frozen vials. To limit the risks of cross-contamination and over-subculturing, all cell lines used in our laboratory are passaged for no more than one month. Cell morphology is monitored several times per week during culture. If there are any changes in cell appearance, growth kinetics or performance in routine assays in our laboratory, cells are discarded and a new culture is established from frozen stocks.

Mycoplasma contamination

The cell lines used tested negative for mycoplasma.

Commonly misidentified lines
(See ICLAC register)

No commonly misidentified cell lines were used.

Animals and other organisms

Policy information about studies involving animals; ARRIVE guidelines recommended for reporting animal research

Laboratory animals

C57BL/6 mice were purchased from Japan SLC.

mFcRn-/- mice (stock number: 003982) and hFcRn Tg 276 homozygote mice (stock number: 004919) were purchased from The Jackson Laboratory, and bred in order to obtain mFcRn-/-, hFcRn Tg 276 heterozygote mice.

Chimaeric mice with humanized livers (PXB-mice, PhoenixBio) were generated from urokinase-type plasminogen activator-cDNA/ severe combined immunodeficiency mice transplanted with human hepatocytes with approximately 80% of hepatocytes being humanized (ref. 48).

Mice were bred and maintained at the Advanced Science Research Center Institute for Experimental Animals, Kanazawa University, or at PhoenixBio Inc.

Wild animals

The study did not involve wild animals.

Field-collected samples

The study did not involve samples collected from the field.

Ethics oversight

All animal experimental procedures were conducted in accordance with the guidelines provided by the Proper Conduct of Animal Experiments (June 1, 2006; Science Council of Japan). The procedures were approved by the Institutional Review Board of Kanazawa

University or Laboratory Animal Ethics Committee of PhoenixBio. The procedures involving humanized liver tissues were approved by Utilization of Human Tissue Ethical Committee of PhoenixBio.

Note that full information on the approval of the study protocol must also be provided in the manuscript.

Flow Cytometry

Plots

Confirm that:

☒ The axis labels state the marker and fluorochrome used (e.g. CD4-FITC).

☒ The axis scales are clearly visible. Include numbers along axes only for bottom left plot of group (a 'group' is an analysis of identical markers).

☒ All plots are contour plots with outliers or pseudocolor plots.

☒ A numerical value for number of cells or percentage (with statistics) is provided.

Methodology

Sample preparation	The binding of peptide-grafted Fc proteins to Met-knockout CHO cells and Met-reconstituted CHO cells (ref. 27) were detected using flow cytometry. Cells were detached from dishes by a brief treatment with 0.025% trypsin and 1 mM ethylenediaminetetraacetic acid, plated at 200,000 cells per well and incubated with peptide-grafted Fc proteins diluted at 10 µg/ml in 100 µl Ham's F-12 medium containing 5% FBS on ice for 1.5 h. After washing twice with ice-cold PBS, cells were incubated with Alexa Fluor 488-labeled goat anti-human IgG (1:400 dilution in 100 µl Ham's F-12 medium containing 5% FBS, Thermo Fisher Scientific, A11013) on ice for 30 min, then analysed on an EC800 system (Sony). The data were analysed with FlowJo software (Tomy Digital Biology).
Instrument	EC800 system (Sony).
Software	FlowJo software (Tomy Digital Biology).
Cell population abundance	the purity of gated populations was generally in the range of 60–90%, as shown in Supplementary Fig. 11.
Gating strategy	Gate on fsc vs. ssc was set to include all cell populations, but excluding debris and dead cells as shown in Supplementary Fig. 11.

☒ Tick this box to confirm that a figure exemplifying the gating strategy is provided in the Supplementary Information.
